# Mitochondrial genome of *Plasmodium vivax/simium* detected in an endemic region for malaria in the Atlantic Forest of Espírito Santo state, Brazil: do mosquitoes, simians and humans harbour the same parasite?

**DOI:** 10.1186/s12936-017-2080-9

**Published:** 2017-10-30

**Authors:** Julyana Cerqueira Buery, Priscila Thihara Rodrigues, Lícia Natal, Laís Camoese Salla, Ana Carolina Loss, Creuza Rachel Vicente, Helder Ricas Rezende, Ana Maria Ribeiro de Castro Duarte, Blima Fux, Rosely dos Santos Malafronte, Aloísio Falqueto, Crispim Cerutti

**Affiliations:** 1 Tropical Medicine Unit, Federal University of Espírito Santo, Vitória, Avenida Marechal Campos, 1468–Maruípe, Vitória, Espírito Santo, 29043-900 Brazil; 2 Department of Parasitology, University of São Paulo, Avenida Professor Lineu Prestes, 1374, Cidade Universitária, São Paulo, 05508-900 Brazil; 3 Tropical Medicine Institute, University of São Paulo, Avenida Doutor Enéas Carvalho de Aguiar, 470, Cerqueira Cesar, São Paulo, 05403-000 Brazil; 4 Laboratory of Mastozoology and Biogeography, Federal University of Espírito Santo, Avenida Fernando Ferrari, 514, Goiabeiras, Vitória, Espírito Santo, 29075-910 Brazil; 5 Nucleus of Entomology and Malacology of Espírito Santo, Health Department of Espírito Santo State, Rua Pedro Zangrandi, 320, Jardim Limoeiro, Serra, Espírito Santo, 29164-020 Brazil; 6 Superintendency for the Control of Endemies (SUCEN), State Secretary of Health of São Paulo, Rua Paula Souza 166, Luz, São Paulo, 01027-000 Brazil

**Keywords:** *Anopheles*, Malaria, Epidemiology, Real-time polymerase chain reaction, DNA, mitochondrial, Sequence analyses, DNA, Zoonoses

## Abstract

**Background:**

The transmission of malaria in the extra-Amazonian regions of Brazil, although interrupted in the 1960s, has persisted to the present time in some areas of dense Atlantic Forest, with reports of cases characterized by particular transmission cycles and clinical presentations. Bromeliad-malaria, as it is named, is particularly frequent in the state of Espírito Santo, with *Plasmodium vivax* being the parasite commonly recognized as the aetiologic agent of human infections. With regard to the spatial and temporal distances between cases reported in this region, the transmission cycle does not fit the traditional malaria cycle. The existence of a zoonosis, with infected simians participating in the epidemiology, is therefore hypothesized. In the present study, transmission of bromeliad-malaria in Espírito Santo is investigated, based on the complete mitochondrial genome of DNA extracted from isolates of *Plasmodium* species, which had infected humans, a simian from the genus *Allouata*, and *Anopheles* mosquitoes. *Plasmodium vivax/simium* was identified in the samples by both nested PCR and real-time PCR. After amplification, the mitochondrial genome was completely sequenced and compared with a haplotype network which included all sequences of *P. vivax/simium* mitochondrial genomes sampled from humans and simians from all regions in Brazil.

**Results:**

The haplotype network indicates that humans and simians from the Atlantic Forest become infected by the same haplotype, but some isolates from humans are not identical to the simian isolate. In addition, the plasmodial DNA extracted from mosquitoes revealed sequences different from those obtained from simians, but similar to two isolates from humans.

**Conclusions:**

These findings strengthen support for the hypothesis that in the Atlantic Forest, and especially in the state with the highest frequency of bromeliad-malaria in Brazil, parasites with similar molecular backgrounds are shared by humans and simians. The recognized identity between *P. vivax* and *P. simium* at the species level, the sharing of haplotypes, and the participation of the same vector in transmitting the infection to both host species indicate interspecies transference of the parasites. However, the intensity, frequency and direction of this transfer remain to be clarified.

**Electronic supplementary material:**

The online version of this article (10.1186/s12936-017-2080-9) contains supplementary material, which is available to authorized users.

## Background

In Brazil, malaria occurs originally across the entire national territory. However, the Amazon region reports 99% of all the cases in the country [1]. Since the 1940s, a national control programme has kept malaria transmission restricted to the northern area. Consequently, in the 1960s and 1970s, the extra-Amazonian region came close to a complete elimination of the disease. Nevertheless, residual transmission persisted in certain areas of dense Atlantic Forest [2]. In the Atlantic Forest, malaria presents at a very low incidence, with cases being mainly related to *Plasmodium vivax* and presenting few clinical symptoms [1–4]. The low incidence and the territorial dispersion of the reported cases provide evidence in favor of the existence of an unrecognized reservoir of parasites. This in turn raises questions regarding the participation of asymptomatic carriers or local simians in the transmission [5]. Genetic similarity between *P. vivax*, which infects humans, and the parasites which infect simians in the Atlantic Forest, named *Plasmodium simium*, further supports the hypothesis of zoonosis. In fact, following the first report of a natural infection of *P. simium* in a human being [5], the identity between *P. simium* and *P. vivax* has been established many times: (1) by studies regarding the CSP protein in the early 1990s [6], (2) by phylogenetic analyses based on sequencing of the cytochrome b gene of the mitochondrial genome [7], (3) by microsatellite polymorphisms [8], (4) again by CSP variations [9], and (5) by Msp-1 gene sequencing [10]. In this context, the recent evidence presented by Brasil et al. [11] of some single nucleotide polymorphisms differentiating *P. simium* from *P. vivax* do not imply their separation in two different species. In the extra-Amazonian region, the term bromeliad-malaria refers to the disease whose vector, recognized as *Anopheles (Kerteszia) cruzii* [12], depends on bromeliads as breeding sites. Molecular and serological evidence presented by different studies has suggested that bromeliad-malaria is highly dependent on human activities carried out close to the forest environment [13–15]. In addition, the occurrence of the disease is sparse, and the outbreaks are rare [16]. Considering the characteristics presented above and the fact that the parasites harboured by local simians are genetically indistinguishable from those found in human blood, the hypothesis of a zoonotic scenario for bromeliad-malaria is strongly supported [17–23]. However, even with a variety of scientific investigations corroborating the zoonosis hypothesis, considerable debate remains regarding the direction of parasite transference. For instance, by comparing the genetic variability in studies based on the Duffy binding protein of erythrocytes collected from simians of the species *Alouatta guariba*, Costa [24] has suggested that the simian parasite originated from its human counterpart. This hypothesis is additionally supported by Rodrigues et al. [25] based on limited genetic variability between *P. simium* and *P. vivax*.

In their study, Brasil et al. [11] suggested the possibility of using particular single nucleotide polymorphisms (SNPs) in order to differentiate between *P. simium* and *P. vivax*, focusing on whole mitochondrial genome sequences. However, differentiating between two variants of the same species is not an easy task. In order to better ascertain the distinctive genetic characteristics of these variants, this study presents the molecular characterization of *P. vivax/simium* based on the sequencing of the mitochondrial genomes of parasites isolated from both human and simian hosts, and, unprecedentedly, from *Anopheles* mosquitoes in an endemic area of the Brazilian Atlantic Forest.

## Methods

### Study area

**Fig. 1:**
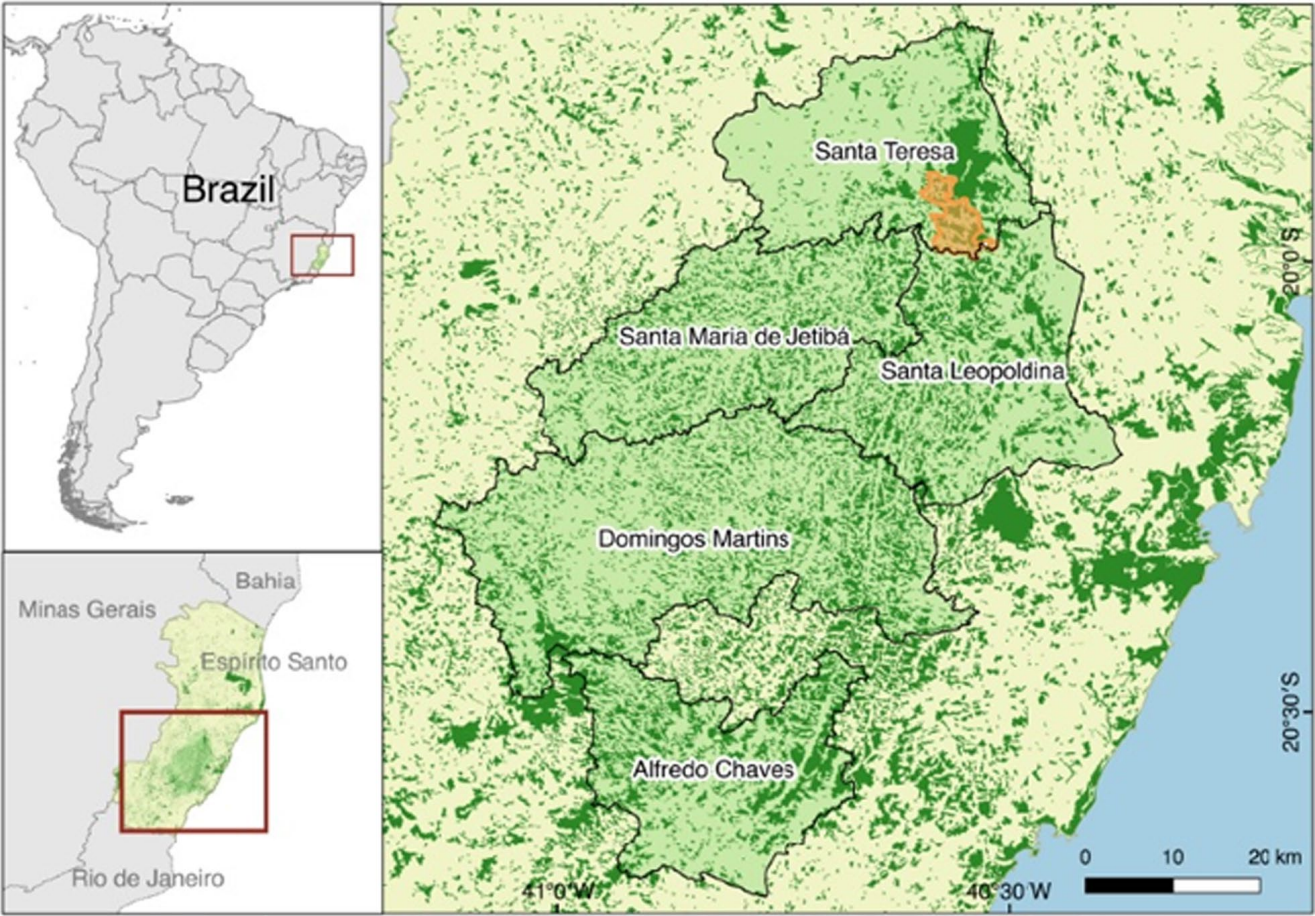
Map showing sampling area of malaria in Santa Teresa municipality, Espírito Santo, Brazil

Espírito Santo is a Brazilian state located in the southeast region, with large areas of dense Atlantic Forest. The fieldwork for collecting samples of anopheline mosquitoes and monkeys was concentrated in Valsugana Velha, district of Santa Teresa, and the main area with reports of malaria in this municipality. Santa Teresa is located 78 km from the capital of Espírito Santo, Vitória (Fig. 1). The landscape in the region is irregular, with a mountainous relief reaching an altitude of 655 m above sea level, and average temperatures that vary between 15.3 and 21.0 °C. Four human blood samples were collected from the inhabitants of Santa Teresa, and 18 from other municipalities of Espírito Santo, also covered by the Atlantic Forest.

### Samples origin

Human blood samples were collected from the 22 cases of malaria caused by *P. vivax* between 2001 and 2004, and previously detected by thick-stained blood smears in the communities of the endemic area [16]. The simian blood sample was obtained from a monkey of the genus *Alouatta* captured alive in Valsugana Velha in 2009. Six specimens of anopheline mosquitoes infected by *P. vivax* and captured in the same area, between 2014 and 2015, were also included (one *Anopheles lutzi*, one *Anopheles strodei* and four *Anopheles cruzii*) [26].

### DNA extraction and confirmation of the infection

Plasmodial DNA from human and simian blood samples was extracted by the QIAamp Blood DNA Mini Kit, while the plasmodial DNA from the mosquitoes was extracted by the DNAeasy Blood and Tissue Kit, both following the instructions of the manufacturer (Qiagen). Infection was confirmed in all samples by nested PCR [27, 28] and real-time PCR (adapted from Rubio et al. [29]) with primers designed to amplify the 18S RNA subunit gene. Positive and negative controls were used in all reactions.

### Amplification and sequencing of the plasmodial complete mitochondrial genome

The complete mitochondrial genome (6 kb) of *P. vivax*/*simium* from the 22 samples of human blood was amplified and sequenced following the protocol proposed by Rodrigues et al. [30]. A new protocol had to be developed in order to perform the amplification of the plasmodial DNA extracted from simian and mosquito samples. Fourteen primers (Pvm1F/Pvm1R to Pvm14F/Pvm14R—Table 1) designed by the software *Primer3* were used in a conventional PCR. The procedure for each sample included 0.5 µl of the enzyme *Taq* DNA polymerase (5.0 U/µl) (Fermentas), 2.0 µl of the extracted DNA, 0.5 µl of each oligonucleotide primer (5.0 µM), 2.0 µl of 10 × Buffer for *Taq* DNA polymerase (with KCl), 0.6 µl of dNTP mix (2.0 mM each) and 1.6 µl of MgCl_2_ (25.0 mM), in a final volume of 20 µl. The amplification was run in the GeneAmp PCR 9700 thermocycler (Applied Biosystems), with initial denaturation at 95 °C for 1 min, followed by 40 cycles of denaturation at 95 °C for 15 s, annealing at 60 °C for 30 s, and extension at 72 °C for 30 min. The final step of the extension was performed at 72 °C for 5 min. PCR products were purified by the Illustra GFX PCR and the Gel Band Purification Kit (GE Healthcare Biosciences), and sequenced using the BigDye kit (Applied Biosystems) in the DNA sequencer ABI 3100 (Applied Biosystems). Complete mitochondrial genome assemblies were generated using the software DNASTAR (version 8.1.13, Madison). The sequences were deposited in GenBank [31]. (Additional file 1: Table S1).

**Table 1:** Sequence of primers for amplifying and sequencing of the complete mitochondrial genome of *P. vivax/simium*

Primer	Sequence (5′–3′)	Size (bp)
*P. vivax/simium*—amplification and sequencing		
Pv mosq mt 1	F:5′-AGCTGTAGATGGATGCTTCG -3′	557
	R:5′-CGAATTGAAGTGTGGAGAGAA -3′	
Pv mosq mt 2	F:5′-TTTCAAGAGTCCAAGGTTCG-3′	577
	R:5′-TGTAACGACTTCCCCATTGT-3′	
Pv mosq mt 3	F:5′-CGTAACCATGCCAACACATA-3′	555
	R:5′-CAGCCTGGGATCAAAAAGTA-3′	
Pv mosq mt 4	F:5′-GACCGTCAAATCCTTTTCATT-3′	584
	R:5′-CGAGAAGGGAAGTGTGTTTC-3′	
Pv mosq mt 5	F:5′-GACCGGTCAAAACGGAAT-3′	537
	R:5′-TTGGAGAATGTTTTGCATCA-3′	
Pv mosq mt 6	F:5′-TGAAAAATGTAAACCTGTAACACAA-3′	589
	R:5′-GTTAACGGCACACAAAATCA-3′	
Pv mosq mt 7	F:5′-TTCCATATAATGATGTTAATGAAGG-3′	544
	R:5′-ATCCATGTCAGGCGTTAAAA-3′	
Pv mosq mt 8	F:5′-AGATCGCGTACTTTGGACTG-3′	599
	R:5′-ACCTCCTCCAAATTCTGCTG-3′	
Pv mosq mt 9	F:5′-TGGTTCTCCAGAACTTGCAT-3′	609
	R:5′-TGAGCCCATACAACACTTCC-3′	
Pv mosq mt 10	F:5′-CCAGCATTTGGTGTTATTAGTC-3′	608
	R:5′-CATCCATTTAAAGCGTCTGG-3′	
Pv mosq mt 11	F:5′-TCTTACCTATGCATTTTCTTGGA-3′	576
	R:5′-CAGTTGCACCCCAATAACTC-3′	
Pv mosq mt 12	F:5′-GCTACAGGTGCATCTCTTGTATT-3′	553
	R:5′-ACCATTCAGGAACAATTTGAA-3′	
Pv mosq mt 13	F:5′-CCCTTCTATCCAAATCTATTAAGTC-3′	596
	R:5′-CTGAATATTCTCTAGCACCAAATG-3′	
Pv mosq mt 14	F:5′-GATTACAGCTCCCAAGCAAA-3′	258
	R:5′-CAACTCCCTATCATGTCTTGC-3′	

### Data analysis

The sequences of the complete mitochondrial genome were aligned by the program ClustalX (version 2.1) and edited manually in the program MEGA (version 7.0). The genetic p-distance between sequences was also calculated in MEGA. Number of haplotype, haplotype diversity (Hd) and nucleotide diversity (Pi) were calculated using DNAsp (version 5). The haplotype network was generated by median-joining [32] in the program *Network*, version 4.6 [33], with standard parameters. Two different datasets were used for the haplotype network analysis: one including all 29 *P. vivax/simium* mitochondrial genome sequences from the Atlantic Forest processed in the present study (n = 29); and another also including all 149 *P. vivax/simium* mitochondrial genome sequences from Brazilian Amazonian and extra-Amazonian samples deposited on GenBank, with 10 of the sequences sampled from simian, and 139 from human mitochondrial DNA (mtDNA) (n = 178).

Bayesian phylogenetic analysis was carried out for *P. vivax/simium* complete dataset (n = 178) using MrBayes (version 3.2.1), with two runs of four chains, three heated and one cold, for 7·10^6^ generations. Only groups with Bayesian posterior probability (BPP) ≥ 95% were considered significant. The consensus tree was visualized using FigTree (version 1.4.2).

Taking the GenBank accession number NC_007243.1 as the reference, the SNPs at positions 4134 and 4468 were observed in all sequences sampled from human, simian and mosquito mtDNA, in order to verify if they were useful in distinguishing between *P. simium* and *P. vivax*. As suggested by Brasil et al. [11], *P. vivax* should present T/A, and *P. simium* C/G in positions 4134 and 4468, respectively.

## Results

**Fig. 2:**
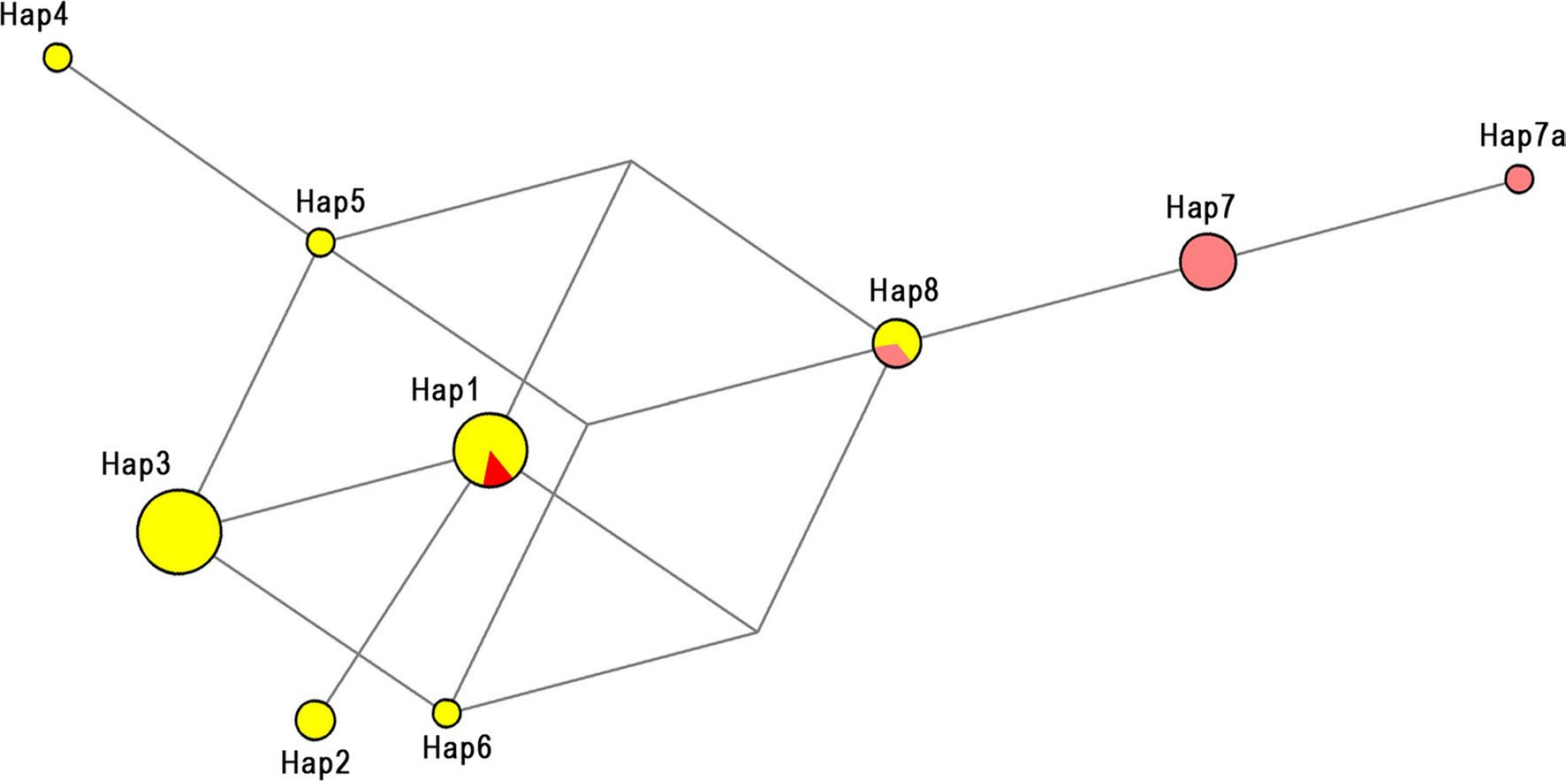
Mitochondrial genome haplotype network of *Plasmodium vivax/simium* sampled in the Atlantic Forest, Espírito Santo, Brazil. Here, 29 samples are presented; 22 from human, 6 from Anopheles mosquitoes and 1 from an *Allouata* monkey

**Fig. 3:**
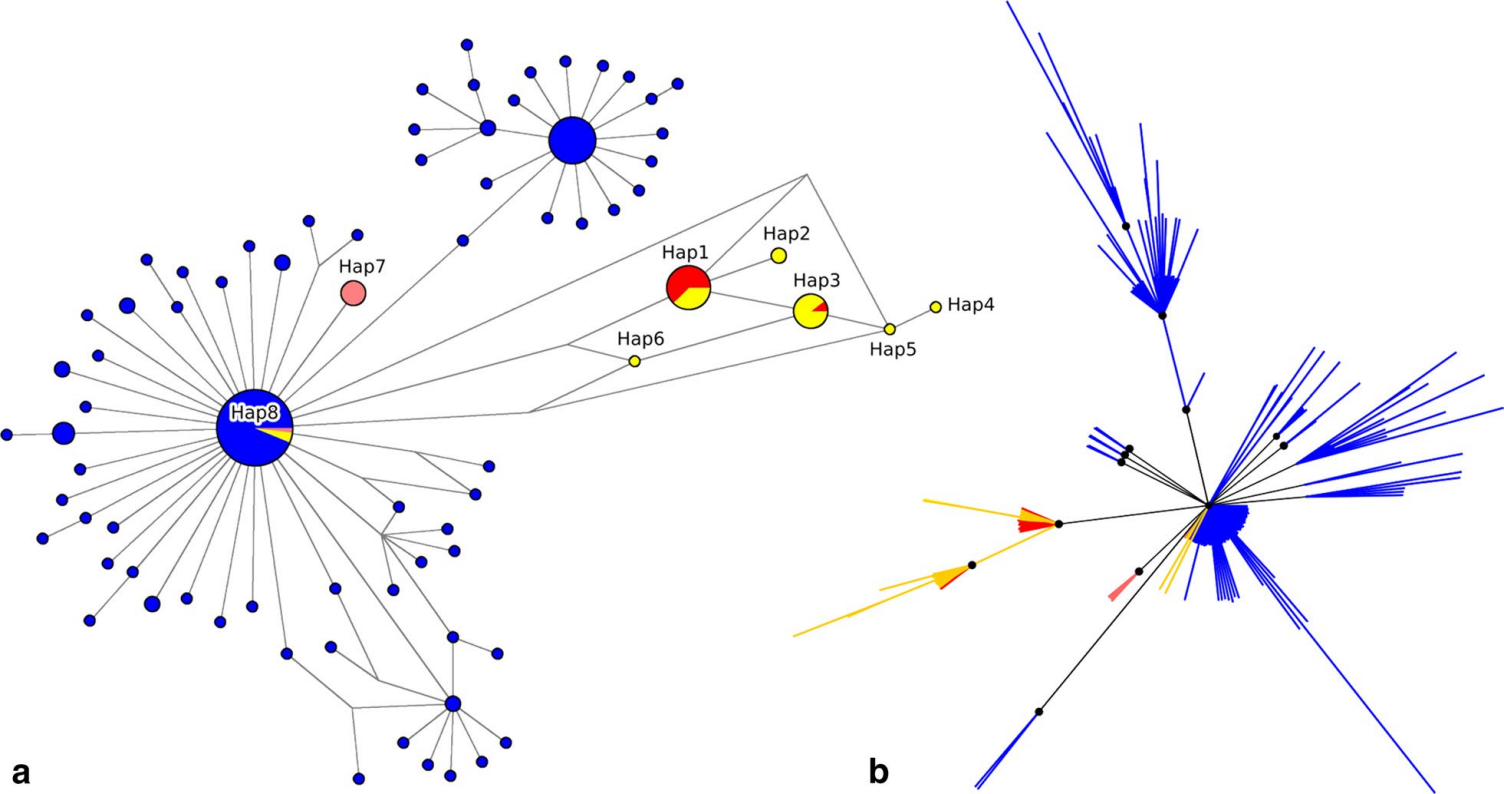
Mitochondrial genome haplotype network and phylogenetic tree of *Plasmodium vivax/simium* from Brazil. 178 samples are presented, including the 29 of Espírito Santo state. **a** The haplotype network by median-joining and **b** the Bayesian phylogenetic tree have the same color pattern, clustered by hosts: blue for human cases from Amazon region; yellow for human cases from Atlantic Forest; red for simian; rose for *Anopheles* mosquitoes. Nodes with Bayesian posterior probabilities ≥ 0.95 are indicated with black circles in the phylogenetic tree

The network comprising only samples from this study (n = 29) is shown in Fig. 2, while the haplotype network and the phylogenetic tree built using the entire dataset (n = 178) are shown in Fig. 3. Among the 22 isolates obtained from human blood from Atlantic Forest inhabitants, seven distinct haplotypes were identified (Figs. 2, 3a: Hap1, Hap2, Hap3, Hap4, Hap5, Hap6 and Hap8). Two of them (Figs. 2, 3a: Hap1 and Hap3) were shared with samples isolated from simians. Hap3 contained SNPs identical to the *P. simium* sequences deposited on GenBank, as shown in Fig. 3a. Hap1 contained SNPs identical to the sequence of the isolate obtained from the simian captured in the study area. Two other samples obtained from the human isolates (Fig. 3a: Hap8) contained SNPs identical to those found in the isolates from human infections acquired in the Amazonian region (*P. vivax*). The remaining four haplotypes (Hap2, Hap4, Hap5 and Hap6) contained SNPs exclusive to the area of the present study (Fig. 3a, Table 2).

**Table 2:** SNPs of the *Plasmodium vivax/simium* mitochondrial genome from human, simian and mosquito samples from Espírito Santo, Brazil

Haplotype	Sample	Source	SNPs (position based on GenBank access NC_007243.1)						
			463	1342	3325	4134^a^	4468^a^	4511	5322
Hap1	PsimiumES	Monkey	T	C	A	C	G	G	A
Hap1	1312MT	Human	–	–	–	–	–	–	–
Hap1	1565MT	Human	–	–	–	–	–	–	–
Hap1	VC57MT	Human	–	–	–	–	–	–	–
Hap1	OJA51_MT	Human	–	–	–	–	–	–	–
Hap1	ACC54_MT	Human	–	–	–	–	–	–	–
Hap1	SV555_MT	Human	–	–	–	–	–	–	–
Hap2	JSB62_MT	Human	–	G	–	–	–	–	–
Hap2	RO54_MT	Human	–	G	–	–	–	–	–
Hap3	GAB847_MT	Human	–	–	T	–	–	–	–
Hap3	1272MT	Human	–	–	T	–	–	–	–
Hap3	1411MT	Human	–	–	T	–	–	–	–
Hap3	1760MT	Human	–	–	T	–	–	–	–
Hap3	1451MT	Human	–	–	T	–	–	–	–
Hap3	FW63MT	Human	–	–	T	–	–	–	–
Hap3	143MT	Human	–	–	T	–	–	–	–
Hap3	111MT	Human	–	–	T	–	–	–	–
Hap3	AJR54_MT	Human	–	–	T	–	–	–	–
Hap4	ALNL53MT	Human	–	–	T	T	–	–	C
Hap5	MA5M61_MT	Human	–	–	T	T	–	–	–
Hap6	761MT	Human	–	–	T	–	A	–	–
Hap7a^b^	479mosq	Mosquito	A	–	–	T	A	C	–
Hap7	485mosq	Mosquito	–	–	–	T	A	C	–
Hap7	632mosq	Mosquito	–	–	–	T	A	C	–
Hap7	343mosq	Mosquito	–	–	–	T	A	C	–
Hap7	260mosq	Mosquito	–	–	–	T	A	C	–
Hap8	1294mosq	Mosquito	–	–	–	T	A	–	–
Hap8	40MT	Human	–	–	–	T	A	–	–
Hap8	103_03MT	Human	–	–	–	T	A	–	–

^a^SNPs suggested by Brasil et al. to differentiate between *P. vivax* (T/A) and *P. simium* (C/G)

^b^Hap7a is represented as Hap7 in the complete data set with 178 samples because position 463 was excluded from the complete database due to missing data in one or more sequences in this site

As demonstrated in Fig. 2, three haplotypes were identified in the isolates obtained from *Anopheles* mosquitoes (Hap7, Hap7a and Hap8): of those, two were exclusive to the vector (Figs. 2, 3a: Hap7 and Hap7a), while the third (Fig. 3a: Hap8) was identical to the haplotype identified in the isolates from the Amazonian region, as well as to two human isolates from the study area (Table 2).

The genetic divergence within the haplotypes sampled from the Atlantic Forest was very low, with only seven SNPs identified from a stretch of DNA 5590 bp long (maximum p-distance 0.1%) (Table 2). Among these, three SNPs were found in the non-coding region of the mitochondrial genome, one SNP within the *CYTB* gene sequence (synonymous mutation), and three SNPs within the *COX1* gene sequence (one synonymous and two nonsynonymous mutations). *COX3* sequences were conserved among all samples from the Atlantic Forest. Furthermore, both haplotype diversity (Hd) and nucleotide diversity (Pi) were low for samples from the Atlantic Forest (Table 3). Interestingly, even though simian isolates represent larger and more geographically widespread samples than mosquito isolates, they had lower Hd and lower Pi (Table 3).

**Table 3:** Number of haplotypes, nucleotide diversity and haplotype diversity in *Plasmodium vivax/simium* mitochondrial genomes sampled from different hosts in Amazonian and Extra-Amazonian regions, Brazil

Source of isolates	Number of isolates	Number of haplotypes	Nucleotide diversity (Pi)	Haplotype diversity (Hd)
Amazonian humans	139	69	0.00047	0.880
Atlantic Forest humans	22	7	0.00023	0.771
Mosquitoes	6	3	0.00011	0.600
Simians	11	2	0.00003	0.182
Total	178	76	0.00051	0.907

The two SNPs suggested by Brasil et al. [11] could not be used to distinguish between *P. simium* and *P. vivax*, at least for the samples of the present study. This is because some haplotypes had sequences different from those proposed as distinctive between *P. simium* and *P. vivax*. More specifically, they showed a combination of these sequences (Table 2: Hap4, Hap5, Hap6). The phylogenetic tree (Fig. 3b) also shows that isolates sampled from humans, mosquitoes and simians were not reciprocally monophyletic and some of these sequences clustered together with high statistical support (BPP ≥ 95%).

The haplotype network showed a reticulate relationship between haplotypes, with no evidence of isolation of any haplotype, and with only one or two mutation steps connecting all of the sequences from the samples of the Atlantic Forest region.

## Discussion

*Plasmodium vivax* is a ubiquitous protozoan with a cosmopolitan distribution, causing infections in a number of populations across different continents. Its South American counterpart, *P. simium,* is the aetiological agent of malaria in simians inhabiting the Atlantic Forest [34]. Several studies have suggested that *P. vivax* and *P. simium* are the same species, based on their genetic similarities [5–10]. In some areas, including the one of the present study, both simians and humans are infected by this agent, making the hypothesis of zoonosis plausible. The results of the present study, uncovering a haplotype diversity in a situation of low genetic divergence in Espírito Santo, indicate a heterogeneity of the isolates obtained from different host species, and strengthen the understanding that *P. vivax* and *P. simium* are the same species with small genetic variations. What is more, these results corroborate the findings of Costa et al. [35] and Rodrigues et al. [25], whose phylogenetic analyses of samples from different world regions indicated a recent transfer of the parasite from humans to New World simians.

Among the eight haplotypes identified in the study area, two were common to humans and simians, based both on the sequences deposited on GenBank, as well as those obtained from the local simian. This finding represents evidence of parasite transmission from one species to another. At the same time, such a sharing could not be confirmed for the remaining haplotypes, as the sequences were clearly distinctive. Four of the haplotypes obtained from humans were exclusive to the study area, and the two remaining ones were compatible with those previously considered from the Amazonian region [25].

The inclusion of samples obtained from mosquito vectors for the comparison of mitochondrial sequences had never been performed before in Brazil, despite being previously suggested by Brasil et al. [11]. Interestingly, the results of the present study were not consistent with Rodrigues et al. [25], who suggested that two SNPs were distinct between malaria from the Atlantic Forest (C/G) and from the Amazonian region (A/T). Rather, plasmodial DNA extracted from mosquito vectors from the Atlantic Forest study area in Espírito Santo revealed the nucleotides A/T at these loci. These same SNPs were also proposed by Brasil et al. [11] as distinctive between *P. vivax* and *P. simium*. However, the present results demonstrate that said SNPs were not able to distinguish between the two lineages in Espírito Santo, as they were not fixed in at least three samples from humans in the study area (Table 2). Furthermore, it was shown that isolates from different hosts share haplotypes, and there is no evidence of monophyly among human and simian samples.

The mosquitoes responsible for the transmission of malaria in the Atlantic Forest system belong to the *Kerteszia* subgenus, with the species *Anopheles cruzii* [26, 36, 37] being the most prominent. Specimens of species of the subgenus *Nyssorhynchus* have also been captured in the region, occasionally infected by *P. vivax*/*simium* recovered from the blood contained in their abdomens [26]. Though it is possible that the mosquitoes of the subgenus *Nyssorhynchus* are being infected by feeding on human blood, their role as vectors is improbable. The fact that haplotype 8 was obtained from humans and from only one pool of *Anopheles (Nyssorhynchus) strodei* captured close to dwellings suggests that the mosquitoes were infected by the humans only incidentally. Haplotype 8 has sequences identical to those previously considered specific to the Amazonian region. Two other haplotypes (Hap7 and Hap7a), obtained from other mosquitoes, despite being closely related to those from the Amazonian region, also have distinctive SNPs, making them exclusive for the mosquitoes of the study area (Figs. 2, 3, Table 2).

The study has some limitations. The parasite DNA was obtained from humans, the simian, and mosquitoes in different periods, precluding any conclusions regarding a possible circulation of all the haplotypes with the same magnitude at the same time. In addition, only a single simian sample was available, preventing determination of the diversity of the haplotypes infecting this host species (Fig. 2). The haplotype network constructed based on ten simians from the Atlantic Forest revealed two different haplotypes shared with humans in the study area (Fig. 3a: Hap1 and Hap3). Araújo et al. [38] highlighted the apparent rareness of simian malaria in the Amazonian region, attributing it to the difficulties in capturing the non-human primates, and in obtaining samples of good quality. The same observational difficulties are applicable to the conditions of the Atlantic Forest.

The genetic diversity found in the present study is greater than that reported by Brasil et al. [11]. The presence of sequences identified in isolates obtained from mosquitoes and shared by two isolates from humans, but different from those obtained from simians in Espírito Santo indicates that the transmission cycle of this residual malaria is complex and cannot be adequately ascertained by only a few studies with small samples. It suggests that interspecies transference of the parasites has either occurred in the past or is still occurring. However, the intensity, frequency and direction of this transfer remain to be clarified.

The merit of the present study, similar to the one by Brasil et al. [11] is to document the presence of the same parasite in both human and simian hosts. Finding the same parasite in two host species is necessary, but not sufficient evidence to confirm a zoonosis. In order to verify a zoonotic cycle, one would have to show different genetic diversities of the parasites between hosts and estimate the time to the most recent common ancestor by phylogenetic analysis of specimens isolated from both hosts. Such an approach was performed to analyse the transmission of *Plasmodium knowlesi*, uncovering a higher number of genotypes per infection in simians than in humans [39]. Additionally, the time to the most recent common ancestor based on the analysis of mtDNA revealed that the species was derived from an ancestral parasite population that existed prior to human settlement in Southeast Asia [39]. Both findings were able to support the hypothesis of an actual zoonosis, pointing to a recent transference of the parasites to the human population. In the case of *P. vivax/simium,* however, the evidence points in the opposite direction. Here, the haplotype diversity is lower among the simians and the phylogenetic analyses indicate a recent transfer of the species from humans to simians [25, 34]. Consequently, the present study, just like the study of Brasil et al. [11] is not sufficient to determine definitively how the transference occurs in the Atlantic Forest, precluding any conclusion regarding a zoonotic cycle.

By including more samples from simians and vectors, all obtained in the same period, future studies should facilitate a deeper understanding of the transmission cycle of this singular endemic disease.

## Conclusions

Sequencing of the complete mitochondrial genome of *P. vivax/simium* in an area of the Atlantic Forest in Brazil uncovered eight haplotypes, two of which were shared by human and simian hosts. Interestingly, the other six haplotypes were distinctive, harboring sequences either unique to human infections in the Atlantic Forest or identical to those of the Amazonian region. Such results indicate the possibility of a zoonotic cycle, but given the observed diversity of the haplotypes, more studies are necessary to better ascertain the dynamics of the transference of parasites between humans and simians.

## Additional file

**Additional file 1: Table S1.** *GenBank* accession numbers of published sequences used to construct the haplotype networks and the phylogenetic tree.

## Abbreviations

DNA: deoxyribonucleic acid
PCR: polymerase chain reaction
SNP: single nucleotide polymorphism
km: kilometers
°C: degrees Celsius
kb: kilobases
µl: microliters
U: unit
µM: micromolars
mM: millimolars
T: thymine
A: adenine
C: cytosine
G: guanine
IBAMA: Brazilian Institute of the Environment and Renewable Natural Resources
SISBIO: Biodiversity Information and Authorization System
Bp: base pair

## References

[1] Pina-Costa A, Brasil P, Di Santi SM, Araújo MP, Suarez-Mutis MC, Santelli ACF (2014). Malaria in Brazil: what happens outside the Amazonian endemic region?. Mem Inst Oswaldo Cruz.

[2] Ministério da Saúde. Situação Epidemiológica da Malária no Brasil, 2000 a 2011. Boletim Epidemiológico. 2013;44. Brasília.

[3] Coura JR, Suarez-Mutis M, Ladeia-Andrade S (2006). A new challenge for malaria control in Brazil: asymptomatic *Plasmodium* infection—a review. Mem Inst Oswaldo Cruz.

[4] Lindblade KA, Steinhardt L, Samuels A, Kachur SP, Slutsker L (2013). The silent threat: asymptomatic parasitaemia and malaria transmission. Expert Rev Anti-Infect Ther..

[5] Deane LM (1992). Simian malaria in Brazil. Mem Inst Oswaldo Cruz.

[6] Goldman IF, Qari SH, Millet PG, Collins WE, Lal AA (1993). Circumsporozoite protein gene of *Plasmodium simium*, a *Plasmodium vivax*-like monkey malaria parasite. Mol Biochem Parasit..

[7] Escalante AA, Freeland DE, Collins WE, Lal AA (1998). The evolution of primate malaria parasites based on the gene encoding cytochrome b from the linear mitochondrial genome. Proc Natl Acad Sci USA.

[8] Leclerc MC, Durand P, Gauthier C, Patot S, Billotte N (2004). Meager genetic variability of the human malaria agent *Plasmodium vivax*. Proc Natl Acad Sci USA.

[9] Lim CS, Tazi L, Ayala FJ (2005). *Plasmodium* vivax: recent world expansion and genetic identity to *Plasmodium simium*. Proc Natl Acad Sci USA.

[10] Tazi L, Ayala FJ (2011). Unresolved *direction of hos*t tr*ansfer of Plasmodium vivax* v. *P. simium and P. malariae* v. *P. brasilianum*. Infect Genet Evol..

[11] Brasil P, Zalis MG, Pina-Costa A, Siqueira AM, Bianco C, Silva S (2017). *Plasmodium simium* causing human malaria: a zoonoses with outbreak potential in the Rio de Janeiro Brazilian Atlantic forest. Lancet Glob Health..

[12] Downs WG, Pittendrigh CS (1946). Bromelian malaria in Trinidad, British West Indies. Am J Trop Med..

[13] Carvalho ME, Glasser CM, Ciaravolo RMC, Etzel A, Santos LA, Ferreira CS (1988). Sorologia de malária vivax no foco Aldeia dos índios, município de Peruíbe, Estado de São Paulo, 1984 a 1986. Cad Saúde Públ..

[14] Curado I, Duarte AMRCD, Lal AA, Oliveira SG, Kloetzel JK (1997). Antibodies anti-bloodstream and circumsporozoite antigens (*Plasmodium vivax* and *Plasmodium malariae/P.brasilianum*) in areas of very low malaria endemicity in Brazil. Mem Inst Oswaldo Cruz..

[15] Curado I, Malafronte RS, Duarte AMRC, Kirchgatter K, Branquinho MS, Galati EAB (2006). Malaria epidemiology in low-endemicity areas of the Atlantic Forest in the Vale do Ribeira, São Paulo, Brazil. Acta Trop..

[16] Cerutti C, Boulos M, Coutinho AF, Hatab C, Rezende HR, Duarte AM (2007). Epidemiologic aspects of the malaria transmission cycle in an area of very low incidence in Brazil. Malar J..

[17] Lal AA, La De, Cruz VF, Collins WE, Campbell GH, Procell PM, McCutchan TF (1988). Circum*sporozoite protein gen*e from *Plasmodium brasilianum*. Animal reservoirs for human malaria parasites?. J Biol Chem.

[18] Arruda ME, Nardin EH, Nussenzweig RS, Cochrane AH (1989). Sero-epidemiological studies of malaria in indian tribes and monkeys of the Amazon Basin of Brazil. Am J Trop Med Hyg.

[19] Fandeur T, Volney B, Peneau C, De Thoisy B (2000). Monkeys of the rainforest in French Guiana are natural reservoirs for *P. brasilianum/P. malariae*. Parasitology.

[20] Volney B, Pouliquen JF, De Thoisy B, Fandeur T (2002). A sero-epidemiological study of malaria in human and monkey population in French Guiana. Acta Trop.

[21] Duarte AM, Porto MAL, Curado I, Malafronte RS, Hoffmann EHE, Oliveira SG (2006). Widespread occurrence of antibodies against circumsporozoite protein and against blood forms of *Plasmodium vivax*, *P. falciparum* and *P. malariae* in Brazilian wild monkeys. J Med Primatol.

[22] Duarte AM, Malafronte RS, Cerutti C, Curado I, Paiva BR, Maeda AY (2008). Natural *Plasmodium* infections in Brazilian wild monkeys: reservoirs for human infections?. Acta Trop.

[23] Yamasaki T, Duarte AM, Curado I, Summa MEL, Dafne VD, Neves A (2011). Detection of etiological agents of malaria in howler monkeys from Atlantic forests, rescued in regions of São Paulo city, Brazil. J Med Primatol..

[24] Costa DC. A infecção malárica pelo *Plasmodium simium/Plasmodium vivax* em primatas não humanos da Mata Atlântica brasileira, 2014. p. 178 [Tese de Doutorado, Centro de Pesquisas René Rachou—Fundação Oswaldo Cruz].

[25] Rodrigues PT, Valdivia HO, Oliveira TC, Alves JMP, Duarte AMRC, Cerutti Jr C, et al. Human migration and the spread of malaria parasites to the New World. bioRxiv. 2017. doi: 10.1101/141853.

[26] Buery JC, Rezende HR, Natal L, Santana LS, Menezes RMT, Fux B, et al. Ecological characterization and infection of Anophelines (Diptera: Culicidae) of the Atlantic Forest in the southeast of Brazil over a 10 year period: has the behaviour of the autochthonous malaria vector changed? bioRxiv. 2017. doi: 10.1101/146803. 10.1590/0074-02760170225 PMC5722266 29236924

[27] Kimura M, Kaneco O, Liuc Q, Zhouc M, Kawamotoc F, Watayad Y (1997). Identification of the four species of human malaria parasites by nested PCR that targets variant sequences in the small subunit rRNA gene. Parasitol Int.

[28] Win TT, Lin K, Mizuno S, Zhou M, Liu Q, Ferreira MU (2002). Wide distribution of *Plasmodium ovale* in Myanmar. Trop Med Int Health..

[29] Rubio JM, Benito A, Roche J, Berzosa PJ, García ML, Micó M (1999). Semi-nested, multiplex polymerase chain reaction for detection of human malaria parasites and evidence of *Plasmodium vivax* infection in Equatorial Guinea. Am J Trop Med Hyg..

[30] Rodrigues PT, Alves JMP, Santamaria AM, Calzada JE, Xayavong M, Parise M (2014). Using mitochondrial genome sequences to track the origin of imported *Plasmodium vivax* infections diagnosed in the United States. Am J Trop Med Hyg.

[31] GenBank. National Center for Biotechnology Information, US National Library of Medicine, Bethesda. 2017. http://www.ncbi.nlm.nih.gov/GenBank. Accessed 17 Oct 2017.

[32] Bandelt H-J, Forster P, Röhl A (1999). Median-joining networks for inferring intraspecific phylogenies. Mol Biol Evol.

[33] Network. Fluxus Technologies, Suffolk. 2017. http://www.fluxus-engineering.com. Accessed 23 Feb 2017.

[34] Deane LM, Ferreira Neto JA, Okumura M, Ferreira MO (1969). Malaria parasites of Brazilian monkeys. Rev Inst Med Trop Sao Paulo.

[35] Costa DC, Assis GMP, Silva FAS, Araújo FC, de Souza Junior JC, Braga Hirano ZM (2015). *Plasmodium simium*, a *Plasmodium vivax*-related malaria parasite: genetic variability of Duffy binding protein II and the Duffy antigen/receptor for chemokines. PLoS ONE.

[36] Rezende HR, Soares RM, Cerutti C, Alves IC, Natal D, Urbinatti PR (2009). Entomological characterization and natural infection of anophelines in an area of the Atlantic Forest with autochthonous malaria cases in mountainous region of Espírito Santo State, Brazil. Neotrop Entomol..

[37] Rezende HR, Falqueto A, Urbinatti PR, De Menezes RM, Natal D, Cerutti C (2013). Comparative study of distribution of anopheline vectors (diptera: culicidae) in areas with and without malaria transmission in the highlands of an extra-Amazonian region in Brazil. J Med Entomol.

[38] Araújo MS, Messias MR, Figueiró MR, Gil LHS, Probst CM, Vidal NM (2013). Natural *Plasmodium* infection in monkeys in the state of Rondônia (Brazilian Western Amazon). Malar J..

[39] Lee KS, Divis PCS, Zakaria SK, Matusop A, Julin RA, Conway DJ (2011). *Plasmodium knowlesi*: reservoir hosts and tracking the emergence in humans and macaques. PLoS Pathog.

