# Supplementary material for: Mitochondrial genome of Plasmodium vivax/simium detected in an endemic region for malaria in the Atlantic Forest of Espírito Santo state, Brazil: do mosquitoes, simians and humans harbour the same parasite?
Source: Malar J. 2017 Oct 30;16:437. doi: 10.1186/s12936-017-2080-9 (PMC5663072; doi:10.1186/s12936-017-2080-9)
Supplement: Supplementary file 1 — Additional file 1: Table S1. GenBank accession numbers of published sequences used to construct the haplotype networks and the phylogenetic tree. [file 12936_2017_2080_MOESM1_ESM.docx]

**Additional file 1: Table.** *GenBank* accession numbers of published sequences used to construct the haplotype networks and the phylogenetic tree.

| **NAME** | **ACCESS NUMBER** | **NAME** | **ACCESS NUMBER** | **NAME** | **ACCESS NUMBER** | **NAME** | **ACCESS NUMBER** | **NAME** | **ACCESS NUMBER** | **NAME** | **ACCESS NUMBER** |
| --- | --- | --- | --- | --- | --- | --- | --- | --- | --- | --- | --- |
| SG77 | AY598097.1 | AD80 | AY598074.1 | Cu12A | KC330568.1 | Ma185B | KC330591.1 | PV571A | KC330614.1 | 485mosq | MF197852 |
| BP1 | AY598096.1 | AD82 | AY598075.1 | Cu16C | KC330569.1 | Ma148A | KC330592.1 | Be399A | KC330615.1 | 632mosq | MF197853 |
| BL79 | AY598095.1 | belem3 | AY791539.1 | Cu12B | KC330570.1 | Ma185C | KC330593.1 | Be372A | KC330616.1 | 345mosq | MF197854 |
| RB69 | AY598094.1 | belem2 | AY791538.1 | Cu48A | KC330571.1 | Ma170B | KC330594.1 | Be309A | KC330617.1 | 260mosq | MF197855 |
| RB68 | AY598093.1 | belem1 | AY791537.1 | Cu72C | KC330572.1 | Ma200A | KC330595.1 | Be399B | KC330618.1 | 1294mosq | MF197856 |
| RB67 | AY598092.1 | 055V | AY791536.1 | Cu64A | KC330573.1 | Ma176D | KC330596.1 | Be309B | KC330619.1 | PsimiumES | MF197850 |
| RB66 | AY598091.1 | 054V | AY791535.1 | Cu94B | KC330574.1 | PV536B | KC330597.1 | Be354A | KC330620.1 | 479mosq | MF197851 |
| RB61 | AY598090.1 | 052V | AY791534.1 | Cu95A | KC330575.1 | PV531A | KC330598.1 | Be372B | KC330621.1 |  |  |
| DE76 | AY598089.1 | 051V | AY791533.1 | Cu6A | KC330576.1 | PV528B | KC330599.1 | Be367C | KC330622.1 |  |  |
| DE75 | AY598088.1 | 050V | AY791532.1 | Cu94C | KC330577.1 | PV536C | KC330600.1 | Be344C | KC330623.1 |  |  |
| BA72 | AY598087.1 | 048V | AY791531.1 | Cu94A | KC330578.1 | PV528A | KC330601.1 | Be334B | KC330624.1 |  |  |
| PC59 | AY598086.1 | 046V | AY791530.1 | Cu95B | KC330579.1 | PV566A | KC330602.1 | Be334A | KC330625.1 |  |  |
| PC51 | AY598085.1 | 045V | AY791529.1 | Cu15.1 | KC330580.1 | PV559A | KC330603.1 | Be324A | KC330626.1 |  |  |
| PC47 | AY598084.1 | Cu72A | KC330558.1 | Ma148B | KC330581.1 | PV537A | KC330604.1 | Be395A | KC330627.1 |  |  |
| PC44 | AY598083.1 | Cu61A | KC330559.1 | Ma166C | KC330582.1 | PV536A | KC330605.1 | Be395B | KC330628.1 |  |  |
| PC43 | AY598082.1 | Cu64B | KC330560.1 | Ma148C | KC330583.1 | PV537C | KC330606.1 | Be344A | KC330629.1 |  |  |
| PC40 | AY598081.1 | Cu48C | KC330561.1 | Ma161A | KC330584.1 | PV566B | KC330607.1 | Be344B | KC330630.1 |  |  |
| PC38 | AY598080.1 | Cu72B | KC330562.1 | Ma166A | KC330586.1 | PV540B | KC330608.1 | Be359A | KC330631.1 |  |  |
| PC35 | AY598079.1 | Cu16A | KC330563.1 | Ma165A | KC330585.1 | PV537B | KC330609.1 | Be359AA | KC330632.1 |  |  |
| AD92 | AY598078.1 | Cu28C | KC330564.1 | Ma158A | KC330587.1 | PV533A | KC330610.1 | Cu16B | KC330567.1 |  |  |
| AD90 | AY598077.1 | Cu29A | KC330565.1 | Ma176A | KC330588.1 | PV533C | KC330611.1 | Ma163A | KC330590.1 |  |  |
| AD89 | AY598076.1 | Cu29B | KC330566.1 | Ma170A | KC330589.1 | PV560A | KC330612.1 | PV540C | KC330613.1 |  |  |

References:

Jongwutiwes S, Putaporntip C, Iwasaki T, Ferreira MU, Kanbara H, Hughes AL. Mitochondrial genome sequences support ancient population expansion in *Plasmodium vivax*. Mol Biol Evol. 2005; 22(8):1733-39.

Mu J, Joy DA, Duan J, Huang Y, Carlton J, Walker J, Barnwell J, Beerli P, Charleston MA, Pybus OG, Su XZ. Host switch leads to emergence of *Plasmodium vivax* malaria in humans. Mol Biol Evol. 2005; 22(8):1686-93.

Taylor JE, Pacheco MA, Bacon DJ, Beg MA, Machado RL, Fairhurst RM, Herrera S, Kim JY, Menard D, Povoa MM, Villegas L, Mulyanto, Snounou G, Cui L, Zeyrek FY, Escalante AA. The Evolutionary History of *Plasmodium vivax* as Inferred from Mitochondrial Genomes: Parasite Genetic Diversity in the Americas. Mol Biol Evol. 2013; 30(9):2050-64.
